# ENGAGING HIGH SCHOOL STUDENTS TO INCREASE RESEARCH-FOCUSED SMARTWATCH USE BY MULTICULTURAL, RURAL OLDER RESIDENTS

**DOI:** 10.1093/geroni/igae098.0917

**Published:** 2024-12-31

**Authors:** Catherine Luna, Lisa Wiese, Bryan Minor, Maureen Schmitter-Edgecombe, Diane Cook

**Affiliations:** Washington State University, Pullman, Washington, United States; Florida Atlantic University, Boca Raton, Florida, United States; Washington State University, Pullman, Washington, United States; Washington State University, Pullman, Washington, United States; Washington State University, Pullman, Washington, United States

## Abstract

At the southern tip of Lake Okeechobee in south central FL is a rural, underresourced, racially/ethnically diverse region. Persons age 65/older (21%) are primarily African American (57%), Hispanic American (29%) and Afro-Caribbean (4%). Health disparities prevalent in the “Glades” region extend to low rates of digital access (60%) and digital literacy (55%). When asking residents “what mattered most” (Boykin & Schoenhofer, 2001), two issues were identified as most important to the older adult stakeholders: increasing technological competence and maintaining brain health. We tested aims developed with the community advisory board, which tapped into a little-used resource: local high school students, who trained older adults in use of smartwatches to monitor exercise, respond to prompts regarding ecological momentary assessments, and complete n-back cognitive assessments. Faith-based health educators facilitated the home-based visits between 33 older residents and 22 students, and collected pre-post written measures addressing technological self-efficacy, cognition, and health literacy. The older adults (91%, 30/33) responded to smartwatch prompts with a 77.80% response rate, which was consistent with current EMA standards: An 80% response rate has been recommended as the target for EMA studies (Turner et al., 2017), and a meta-analysis reported 75% in the pooled response rate (Jones et al., 2019). Of interest was that self-reported cognition and the Mini-Cog scores were not related (Pearson’s correlational analysis) to number of smartwatch prompts answered or health literacy. Six categories of recommendations ranging from student concerns to resident suggestions emerged from this work, which will be shared in this presentation.

